# Life‐threatening and life‐saving inappropriate implantable cardioverter defibrillator shocks

**DOI:** 10.1002/ccr3.893

**Published:** 2017-03-08

**Authors:** Jordi Pérez‐Rodon, David Doiny, Berta Miranda, Nuria Rivas‐Gandara, Ivo Roca‐Luque, Jaume Francisco‐Pascual, Rosa Maria Lidón, David García‐Dorado, Angel Moya Mitjans

**Affiliations:** ^1^ Department of CardiologyArrhythmia UnitHospital Universitari Vall d'HebrónUniversitat Autònoma de BarcelonaEdifici Annexos, planta 9, Passeig Vall d'Hebrón, 119‐12908035BarcelonaSpain; ^2^ Department of CardiologyHospital Universitari Vall d'HebrónUniversitat Autònoma de BarcelonaEdifici Annexos, planta 9, Passeig Vall d'Hebrón, 119‐12908035BarcelonaSpain

**Keywords:** Atrial fibrillation, implantable cardioverter defibrillator, inappropriate shock, lead dislodgement, ventricular fibrillation

## Abstract

An implantable cardioverter defibrillator (ICD) lead dislodgement into the right atrium is a dangerous situation, particularly in patients in atrial fibrillation because atrial fibrillation can be sensed as ventricular fibrillation and true ventricular fibrillation induced with an inappropriate shock. In the presence of shocks, ICD interrogation should be performed as soon as possible.

## Introduction

The implantable cardioverter defibrillator (ICD) is the standard of care for secondary prevention in patients with previous cardiac arrest and for primary prevention in appropriately selected patients with structural heart disease 1, 2, 3. Despite the benefit in terms of survival, the ICD is cause of important morbidity and decrease in quality of life in some patients. Device infection, lead dislodgement, recalls from the industry needing reoperation, and inappropriate therapies are some of the adverse events following ICD implantation 4. In this manuscript, we describe the case of a patient that received six inappropriate ICD shocks that seriously threatened her life.

## Case Report

A 65‐year‐old female patient with a history of prosthetic mechanical mitral valve replacement due to rheumatic heart disease and long‐standing persistent atrial fibrillation (AF) was implanted with a single‐chamber ICD as a secondary prevention, 1 month after a fully recovered cardiac arrest due to ventricular fibrillation (VF). A coronary angiography ruled out severe coronary disease. An echocardiogram documented preserved left ventricular ejection fraction with a mild to moderate prosthetic leak, moderate tricuspid regurgitation, and severe left atrium enlargement. An active fixation lead was used and positioned in the septo‐apical right ventricular area. The intra‐ and postoperative lead parameters were within normal ranges. The ICD was programmed as follows: one zone—VF zone—with detection at 200 bpm and therapy with six shocks (the first one set to 15J and the following five set to maximum‐energy‐36J). The intra‐operative lead position was confirmed by a chest X‐ray predischarge. Testing of ventricular lead 1 week after the implant was also within normal range for threshold, impedance, and R‐wave sensing.

The patient was admitted to the emergency department 3 months later after receiving six consecutive shocks. She denied any cardiac symptoms before the event. The patient referred suffered sudden loss of consciousness immediately after the first shock. A direct witness described that she remained unconscious until at least two more shocks were delivered. Thereafter, three more shocks followed while the patient was fully conscious.

**Figure 1:**
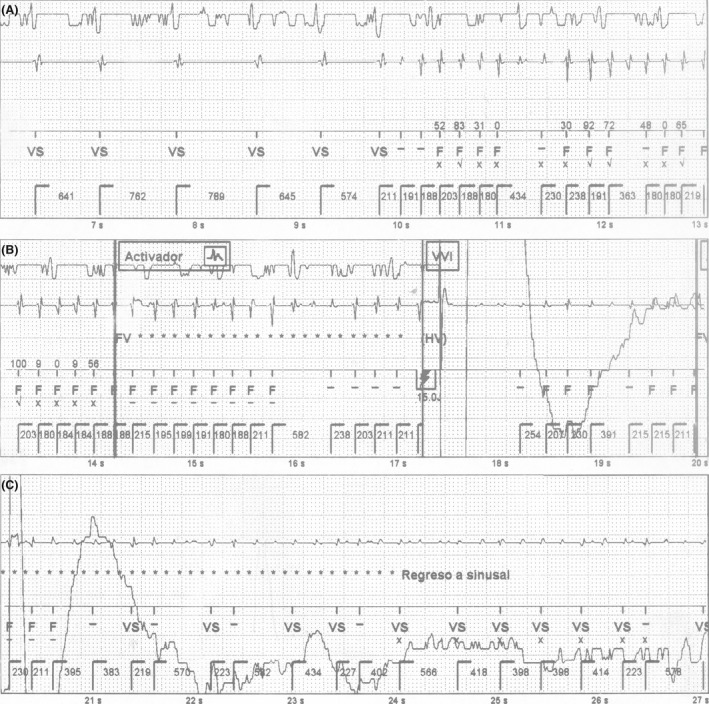
EGMs of the episode are shown in consecutive strips. The upper channel shows the far‐field, the middle one corresponds to the near‐field (ventricular tip‐ ventricular ring), and the lower one to the markers. Panel A: A sudden shortening in basal cycle length that reach VF zone is shown in the near‐field channel, while the far‐field channel remains in the patient´s basal rhythm. Panel B: VF detection criteria are met, and an unsynchronized shock is delivered inducing VF (change in far‐field channel). Panel C: VF is registered in the far‐field channel, while AF is undersensed in the near‐field and a subsequent shock is canceled. Activador = VF therapy activated and start of charging, EGMs = intracardiac electrograms, F = ventricular fibrillation detection, FV = VF detection criteria met, Regreso a sinusal = sinus rhythm restored, VF = ventricular fibrillation, VS = ventricular sensing.

**Figure 2:**
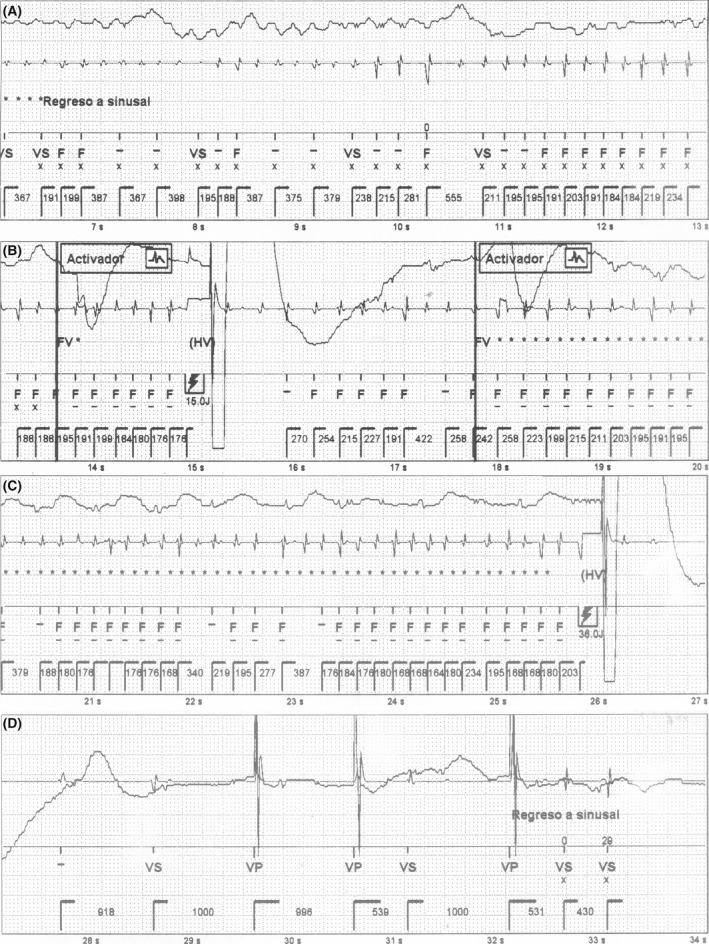
EGMs of the episode are shown in consecutive strips. The upper channel shows the far‐field, the middle one corresponds to the near‐field (ventricular tip‐ ventricular ring), and the lower one to the markers. Panel A: VF is shown in far‐field channel, while unstable low signals are registered in the near‐field channel. Panel B: A stable period of VF detection that fulfilled diagnosis criteria led to a shock that limits VF (see far‐field channel). Panels C and D: Redetection of AF in the near‐field channel within the VF zone induces a shock that restores sinus rhythm. Activador = VF therapy activated and start of charging, EGMs = intracardiac electrograms, F = ventricular fibrillation detection, FV = VF detection criteria met, VF = ventricular fibrillation VP = ventricular pacing, VS = ventricular sensing, Regreso a sinusal = sinus rhythm restored.

**Figure 3:**
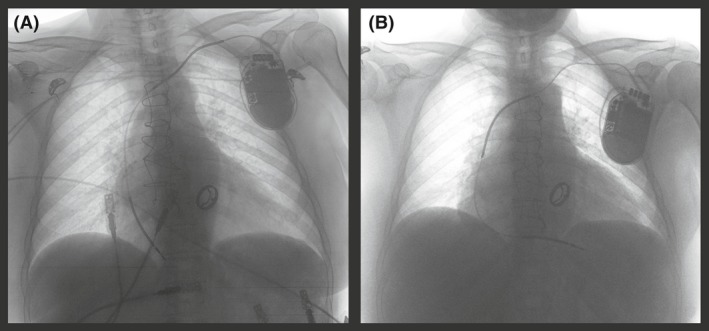
The panel A shows the chest X‐ray in the emergency department. The ventricular lead is dislodged into the right atrium. The panel B corresponds to the routine X‐ray after repositioning the ICD lead.

The stored intracardiac electrograms (EGMs) of the event are shown in Figures 1 and 2. The analysis of the episode revealed a sudden change to rapid and irregular sensed activity in the bipolar ventricular channel, that reached VF zone. At the same time, the far‐field channel showed persistence of patient′s basal rhythm (AF with controlled ventricular response) (Fig. 1). This could only be explained by cross talk sensing of atrial signal during AF in the bipolar ventricular lead, probably due to lead dislodgement. An unsynchronized shock was delivered, and VF was induced. This was documented by the far‐field channel (Fig. 1), which explains the sudden loss of consciousness immediately after the discharge. Then, during a 20‐sec period, the rapid and irregular atrial activity was alternatively sensed and undersensed by the ventricular lead due to low signals, coexisting with VF that was still registered by the far‐field channel (Fig. 1). Undersensing of low signals in the ventricular lead led to delay a subsequent shock. Providentially, it was followed by a stable AF sensing period in the ventricular lead that led, initially to a second ineffective shock (not shown), and subsequently to a third inappropriate but life‐saving shock, that defibrillated VF (Fig. 2). Finally, the device still detecting rapid atrial electrical activity delivered a fourth shock that restored sinus rhythm (Fig. 2). Posteriorly, AF recurred and the device delivered two more inappropriate shocks (not shown) that effectively restored sinus rhythm. The patient was admitted and a chest X‐ray confirmed dislodgement of the ventricular lead in the right atrium (Fig. 3).

A second intervention was scheduled and consequently performed. The ventricular lead was positioned into the right ventricular apex and firmly fixed to ensure its stability. During the follow‐up, the patient remained uneventful without neurological sequelae.

## Discussion

The most common causes of inappropriate ICD therapies are supraventricular tachycardia, especially AF or atrial flutter with rapid ventricular response, T‐wave oversensing, and lead dysfunction (noise and myopotentials) 5. Inappropriate therapies are associated with an adverse effect on health outcomes, quality of life, and mortality 6, 7, 8, but direct fatal proarrhythmia has been occasionally published 9, 10, 11.

In this case, lead dislodgment into the right atrium with AF led to inappropriate unsynchronized shock that induced VF. This mechanism has been previously reported as a fatal mechanism of inappropriate therapy 9. Low AF signals could be undersensed and start anti bradycardia pacing while VF. However, in this case, intermittent sensing of atrial signal during AF led to a subsequent shock that terminated VF and a following discharge restored sinus rhythm and limited inappropriate therapies. To our knowledge, this represents the first case documenting both life‐threatening and life‐saving inappropriate therapies secondary to AF oversensing.

Our report underlines the importance of an ICD interrogation as soon as possible after a shock. In a setting of a dislodged lead, the patient should be continuously monitored and the ICD should be immediately inactivated until the lead is repositioned.

## Authorship

JPR: conceived and designed the study. JPR and DD: drafted the manuscript. BM: designed the figures. NRG, IRL, and JFP: acquired the data. RML, DGD, and ÀMM: made critical revision of the manuscript for key intellectual content.

## Conflict of Interest

None declared.
